# Excess hepsin proteolytic activity limits oncogenic signaling and induces ER stress and autophagy in prostate cancer cells

**DOI:** 10.1038/s41419-019-1830-8

**Published:** 2019-08-09

**Authors:** Ramona Willbold, Katharina Wirth, Thomas Martini, Holger Sültmann, Christian Bolenz, Rainer Wittig

**Affiliations:** 1 Biology group, Institute for Laser Technologies in Medicine and Metrology (ILM) at the University of Ulm, Helmholtzstr. 12, 89081 Ulm, Germany; 2 Dept. of Urology, Ulm University Hospital, Albert-Einstein-Allee 23, 89081 Ulm, Germany; 3 Cancer Genome Research, German Cancer Research Centre (DKFZ) and German Cancer Consortium (DKTK), Im Neuenheimer Feld 280, 69120 Heidelberg, Germany

**Keywords:** Oncogenes, Urological cancer

## Abstract

The serine protease hepsin is frequently overexpressed in human prostate cancer (PCa) and is associated with matrix degradation and PCa progression in mice. Curiously, low expression of hepsin is associated with poor survival in different cancer types, and transgenic overexpression of hepsin leads to loss of viability in various cancer cell lines. Here, by comparing isogenic transfectants of the PCa cell line PC-3 providing inducible overexpression of wild-type hepsin (HPN) vs. the protease-deficient mutant HPN^S353A^, we were able to attribute hepsin-mediated tumor-adverse effects to its excess proteolytic activity. A stem-like expression signature of surface markers and adhesion molecules, Notch intracellular domain release, and increased pericellular protease activity were associated with low expression levels of wild-type hepsin, but were partially lost in response to overexpression. Instead, overexpression of wild-type hepsin, but not of HPN^S353A^, induced relocalization of the protein to the cytoplasm, and increased autophagic flux in vitro as well as LC3B punctae frequency in tumor xenografts. Confocal microscopy revealed colocalization of wild-type hepsin with both LC3B punctae as well as with the autophagy cargo receptor p62/SQSTM1. Overexpression of wild type, but not protease-deficient hepsin induced expression and nuclear presence of CHOP, indicating activation of the unfolded protein response and ER-associated protein degradation (ERAD). Whereas inhibitors of ER stress and secretory protein trafficking slightly increased viability, combined inhibition of the ubiquitin-proteasome degradation pathway (by bortezomib) with either ER stress (by salubrinal) or autophagy (by bafilomycin A1) revealed a significant decrease of viability during overexpression of wild-type hepsin in PC-3 cells. Our results demonstrate that a precise control of Hepsin proteolytic activity is critical for PCa cell fate and suggest, that the interference with ERAD could be a promising therapeutic option, leading to induction of proteotoxicity in hepsin-overexpressing tumors.

## Introduction

The hepsin gene (HPN) is one of the most frequently and prominently overexpressed genes in PCa^1^. Its prostate-specific overexpression in mice causes matrix degradation and—subsequent to additional activation of the SV40 large T-antigen—the formation of metastasizing tumors^2^. HPN encodes for a serine protease of the type II transmembrane class (TTSP), which additionally features a short N-terminal cytoplasmic domain, and a scavenger receptor cysteine rich domain^3^. Subcellular localization studies draw an inconsistent picture and indicate tissue-specific functions of hepsin. Initially identified as cell surface protein critical for cell growth^4^, researchers subsequently described colocalization with desmosomal complexes in ovarian cancer^5^ as well as in mouse mammary epithelial cells (MMEC). In MMEC, loss of the LKB1 tumor suppressor gene induces enhanced expression of hepsin as well as its translocation from desmosomal structures to the cytoplasm, which coincides with the degradation of extracellular matrix (ECM) components (Laminin-332, Collagen IV, Nidogen) and disruption of epithelial integrity^6^. In a mammary gland model, hepsin was found to downregulate its inhibitor hepatocyte growth factor (HGF) activator inhibitor type 1 (HAI-1), leading to enhanced proteolytic activity, activation of cancer-relevant targets, and disruption of desmosomal and hemidesmosomal structures, finally resulting in a loss of epithelial cohesion^7^. In summary, hepsin appears as a modulator of epithelial integrity, and its deregulated and/or mislocalized expression contributes to disturbance of the barrier function of epithelia. Hepsin exhibits pericellular proteolytic activity. In the context of PCa, pro-HGF, and pro-urokinase-type plasminogen activator (pro-uPA) are substrates of particular interest due to their tumor-promoting functions. Both are cleavable in vitro by hepsin and therefore are considered to promote metastasis in hepsin-positive tumors^8–10^.

During overexpression of hepsin in tumor cell lines, adverse effects such as growth suppression, increased cell death, and reduction of invasive growth were observed^11–13^. In patients, immunohistochemical analyses revealed significantly reduced to absent expression of hepsin protein in PCa metastases when compared with primary PCa and its precursor lesion, high-grade prostate intraepithelial neoplasia^14^. Low, but not high expression of hepsin is associated with poor survival in breast cancer^15^, renal cell carcinoma^16^, and hepatocellular carcinoma^17^. Based on these data, researchers coined the term “hepsin-paradox”, which describes the importance of a precise temporal and spatial restriction of hepsin overexpression for the tumor in order to avoid adverse effects^18^. In a recent study using engineered PC-3 PCa cells for inducible overexpression of a hepsin transgene (designated PC3L1-HPN) we reproduced hepsin-induced growth suppression in vitro. We found a dose dependence, i.e., that the extent of adverse effects correlated with expression strength of hepsin. Growth reduction was accompanied by reduced phosphorylation of AKT at Ser^473^ at high hepsin expression levels. Interestingly, this phenotype could not be reverted by exposure to the broad spectrum serine protease inhibitor 4-(2-Aminoethyl)-benzensulfonyl fluoride (AEBSF), but partially reverted by growth on ECM derived from prostate epithelial cells, which suggests that the microenvironment influences viability parameters of hepsin-positive prostate cancer cells^19^.

The present study sought clarification on the role of proteolytic activity for adverse effects during hepsin overexpression. To this end, we constructed a protease-deficient hepsin mutant cDNA (HPN^S353A^) by exchanging the serine residue in the catalytic triad by alanine^20^. The cDNA was site specifically inserted into the PC3L1 clone, giving rise to PC3L1-HPN^S353A^, a cell line isogenic to PC3L1-HPN. Side-by-side analyses using these two cell lines revealed a distinguished role for hepsin proteolytic activity for the “hepsin-paradox” phenotype.

## Materials and methods

### Reagents

Antibodies utilized in this study were from R&D Systems, (Abingdon, UK) (AF4776 sheep polyclonal anti-hepsin), Abcam (Cambridge, UK) (ab6276 mouse monoclonal anti beta-actin), Cosmo Bio (Carlsbad, CA, U.S.A.) (BAM-73-500-EX mouse anti-ATF6), Cell Signaling (Frankfurt, Germany) (9272 rabbit polyclonal anti-AKT, 4060 rabbit monoclonal anti-AKT^Ser473^, 4056 rabbit monoclonal anti-AKT^Ser308^, 2421 rabbit polyclonal anti-cleaved Notch1, 3126 rabbit monoclonal anti phospho-met [Tyr1234/1235], 8198 rabbit monoclonal anti met, 3288 rabbit monoclonal anti-EEA1, 3868 rabbit monoclonal anti-LC3B, 3750 rabbit polyclonal anti integrin alpha6, 2895 mouse monoclonal anti-CHOP), Santa Cruz (Heidelberg, Germany) (sc-19622 rat monoclonal anti integrin alpha6, sc-53352 mouse monoclonal anti integrin alpha2, sc-2473 donkey anti-sheep IgG-HRP, sc-2031 goat anti-mouse IgG-HRP, sc-2030 goat anti-rabbit IgG-HRP), Sigma-Aldrich (Schnelldorf, Germany) (WH0008878M1 mouse monoclonal anti-SQSTM1) and Life Technologies (Darmstadt, Germany) (A11015 donkey anti-sheep IgG Alexa488, A11029 goat anti-mouse IgG Alexa488, A21245 goat anti-rabbit IgG Alexa647). Cell culture media and supplements, Accutase solution, Gateway cloning reagents, chemically competent E. coli DH5α, the Phusion site directed mutagenesis kit, tetramethylrhodamine (TRITC)-labeled phalloidin, and 4′,6-Diamidino-2-Phenylindole (DAPI) were from Life Technologies (Darmstadt, Germany). Fetal bovine serum was from Biochrom (Berlin, Germany). Hygromycin was from Roth (Karlsruhe, Germany), doxycycline (dox), salubrinal, and eeyarestatin-1 were from Sigma-Aldrich, and bafilomycin A1 was from Enzo Life Sciences (Lörrach, Germany). Nitrocellulose membrane and ECL hyperfilm were from GE Healthcare Life Sciences (Freiburg, Germany), ECL western blot detection reagents from Applichem (Darmstadt, Germany). Bortezomib and UK122 were from Santa Cruz, the uPA activity assay kit was from Merck Millipore (Darmstadt, Germany), the Cell Titer Blue cell viability assay was from Promega (Mannheim, Germany), and the Annexin V-FITC conjugate was from Miltenyi (Bergisch-Gladbach, Germany).

### Cell culture

Cells were cultivated using standard conditions (37 °C, 5% CO_2_, >95% humidity). Genetic engineering of derivatives of the PC-3 PCa cell line (ATCC no. CRL-1435) and of the Flp-in T-REx293 cell line (Life Technologies) used in this study was described previously^19^. Cells were cultivated in RPMI (PC-3 and LNCaP) and DMEM high glucose (Flp-in T-REx293) media containing GlutaMAX, 10% fetal calf serum, and penicillin (100 U/ml)/streptomycin (100 µg/ml), respectively. Target gene expression was induced using dox. Cells were routinely checked for mycoplasma contamination. The identity of LNCaP cells and the PC3L1 acceptor cell line was verified by MCA profiling at the Genomics and Proteomics Core Facility of the German Cancer Research Centre (Heidelberg, Germany)^21^.

### PCR-mutagenesis, cloning, and transfection

A full length hepsin cDNA cloned in the Gateway ENTRY vector pENTRY221 (accession number DQ892119) was subjected to site-specific mutagenesis using the Phusion site directed mutagenesis kit according to the manufacturer’s instructions. Briefly, 5′ phosphorylated oligonucleotide primers (for: ggc gac gcc ggt ggt ccc ttt g; rev: ctg gca ggc atc aat gcc acc ctc) were utilized to amplify the plasmid and simultaneously modify codon 353 in the catalytic centre of the enzyme (agc, encoding for serine, to gcc, encoding for alanine), giving rise to the HPN^S353A^ cDNA which encodes for a protease-deficient mutant. Subsequent to intramolecular ligation and transformation, the plasmid was amplified in E. coli DH5α host cells. The mutation was verified by sequencing. Via a Gateway LR reaction, the HPN^S353A^ cDNA was transferred to a proprietary expression vector containing a Flp-recombinase target site (FRT) as well as the tetracycline (tet) repressor gene and a corresponding tet-responsive promoter upstream of the target gene for inducible expression^22^. This vector was then cotransfected with a plasmid encoding for Flp-recombinase into PC3L1, a preselected subclone of the PC-3 prostate carcinoma cell line which harbors a FRT site in the genome for site-specific integration. Hygromycin selection enabled the isolation of recombinant clones (PC3L1-HPN^S353A^) which are isogenic to the previously described PC3L1-HPN clonal cell line^19^.

### Viability assay

Viability assays were conducted using the Cell Titer Blue assay as described previously^19^. Briefly, cells were seeded in triplicates and grown in presence of the different concentrations of dox to induce the target gene. If applicable, drugs were added as indicated. Seventy-two hours post seeding, cells were assayed according to the instructions of the manufacturer. Resorufin-mediated fluorescence (excitation: 544 nm, detection: 590 nm), the intensity of which correlates to the number of viable cells per well, was measured in the Fluostar Omega microplate reader (BMG Labtech, Ortenberg, Germany). Subsequent to blank correction, viability was calculated as percentage of the untreated control, respectively. At least three independent experiments were performed in triplicate.

### Acridine orange staining

Upon excitation with blue light (*λ* = 488 nm), acridine orange (AO)-mediated fluorescence emission spectrum depends on the pH of the cellular environment and thus can be utilized for labeling of acidic compartments such as endosomes, lysosomes, and vacuoles. Within the cell, AO is intercalated into nucleic acids (resulting in green fluorescence), as well as protonated and accumulated in acidic vesicles (resulting in orange fluorescence). The fluorescence ratio orange vs. green is indicative for the acidic vesicle content of a given cell, which may be used for the analysis of lysosomal membrane permeabilization (LMP) or autophagy^23^. Adherent cells were incubated with AO (10 µM, 30 min at 37 °C). Subsequently, the dye was replaced by complete growth medium, and the cells were further cultivated for 30 min under standard conditions and subjected to live cell imaging. For flow cytometry, cells were detached using accutase solution. Subsequent to centrifugation and washing, cells were resuspended in flow cytometry buffer (1% BSA in PBS) containing 0.5 µg/ml DAPI.

### Flow cytometry

For flow cytometry-based analysis of surface marker expression, cells were grown for 48–72 h in the presence of different concentrations of dox for target gene induction. Cells were then harvested using accutase solution, collected by centrifugation and incubated with primary antibodies for 30 min on ice. Subsequent to washing, cells were incubated with secondary antibodies for 30 min on ice. After an additional washing step, cells were resuspended in flow cytometry buffer (1% BSA in PBS) containing propidium iodide (PI, 1 µg/ml) for identification of dead cells. Cell suspensions were measured using a CyFlow Space cytometer (Sysmex-Partec, Münster, Germany). A total of 25,000 events per sample were captured, and data analysis was performed using Flomax software (Version 3.0, Sysmex-Partec). Color compensation was conducted using an algorithm implemented in the software, and dead cells and doublets were excluded from the analysis by gating based on FSC, SSC, and PI-mediated fluorescence. For AO stained cells, flow cytometry was used to quantify the content of acidic vesicles per cell (red/green fluorescence intensity ratio or acidic vesicle index), which was determined using the ratio calculation function in Flomax. AO-mediated fluorescence was excited using the 488 nm laser, and emission was detected in the green range by a 536/40 nm bandpass filter, as well as in the orange–red range using a 620/20 nm bandpass filter. A total of 50.000 events per sample were captured, and dead cells and doublets were excluded from the analysis by gating based on FSC, SSC, and DAPI-mediated fluorescence. For flow cytometric evaluation of apoptosis, cells were stained with Annexin V-FITC conjugate and DAPI as recommended by the manufacturer.

### Cell staining and confocal laser scanning microscopy

Confocal laser scanning microscopy was conducted using a Leica TCS SP8 system (Leica Microsystems, Wetzlar, Germany). Cells were seeded in ibitreat dishes (ibidi, Munich, Germany) and grown for 72 h. For immunofluorescence staining, cells were fixed using 4% PBS-buffered formaldehyde and subsequently stained with primary and secondary antibodies using standard protocols. Counterstaining of filamentous actin and nuclei was carried out using phalloidin-TRITC and DAPI, respectively. Excitation of fluorophores was achieved using integrated lasers emitting at 405 nm (for DAPI), 488 nm (for secondary antibodies labeled with Alexa488), 552 nm (for phalloidin-TRITC) and 638 nm (for secondary antibodies labeled with Alexa647). For AO live cell imaging, excitation of the dye was performed using the 488 nm laser. Neutral compartments of the cell were detected by collecting emitted light at 493–547 nm (designated as “AO neutral”), whereas acidic compartments were detected by collecting emitted light at 575–739 nm (designated as “AO acidic”), respectively. Fluorescence was detected using a HP CL APO 63 × /1.40 OIL CS2 oil immersion objective (Leica Microsystems) and a pinhole setting of 1 Airy unit.

### Urokinase-type plasminogen activator (uPA) activity assay

The uPA activity assay (Chemicon ECM600, Merck Millipore, Darmstadt, Germany) was performed as recommended by the manufacturer. Briefly, cells were seeded in quadruplicate in 96-well plates at 10.000/well and grown in presence (500 pg/ml) vs. absence of dox for 72 h. Subsequently, a chromogenic substrate was added and the blank-corrected absorbance at 405 nm was determined periodically over a time range of 4 h during incubation at 37 °C in a Fluostar Omega microplate reader (BMG Labtech, Ortenberg, Germany). Replicate control experiments in presence of the uPA-specific inhibitor UK122^24^, which was added at 50 µM 48 h post seeding, revealed a significant decrease of absorbance for all corresponding samples, indicating specificity of the reaction. The experiment was conducted twice and resulted in similar datasets, one of which is presented in Fig. 3.

### Western blot analysis

Western blot analysis was performed as described previously^19^. Briefly, cell lysates were subjected to SDS PAGE and subsequently blotted to nitrocellulose membranes. Proteins were specifically detected by using the antibodies listed in the reagents section. Horseradish peroxidase activity was detected using ECL western blotting reagent and ECL hyperfilm.

### Chicken chorioallantoic membrane (CAM) assay

The CAM assay was performed as described previously^19^. Briefly, PC3L1-HPN and PC3L1-HPN^S353A^ cells were pretreated with dox (100 ng/ml) or vehicle (PBS) 96 h prior to transplantation. A total of 7 × 10^5^ cells of each pretreated cell population were seeded in a RPMI1640 medium/Matrigel solution (1:1) to the CAM at embryonic development day (EDD) 8, followed by topical administration of 100 ng/ml dox or vehicle (RPMI1640 medium) at EDD 9. Tumors were grown for additional 72 h. At EDD 12 tumors were sampled, fixed, and subjected to immunohistochemical staining.

### Statistical analysis

For viability assays, significance levels were calculated by considering all data points generated for one sample (technical/biological replicates) and by using the unpaired t-test and the Holm–Sidak method for correction of multiple comparisons in the Graph Pad Prism Software (version 7.05). Samples exhibiting *P* values < 0.05 were considered to be significantly different.

## Results

### Hepsin protein expression in isogenic PC-3 transfectants

**Fig. 1:**
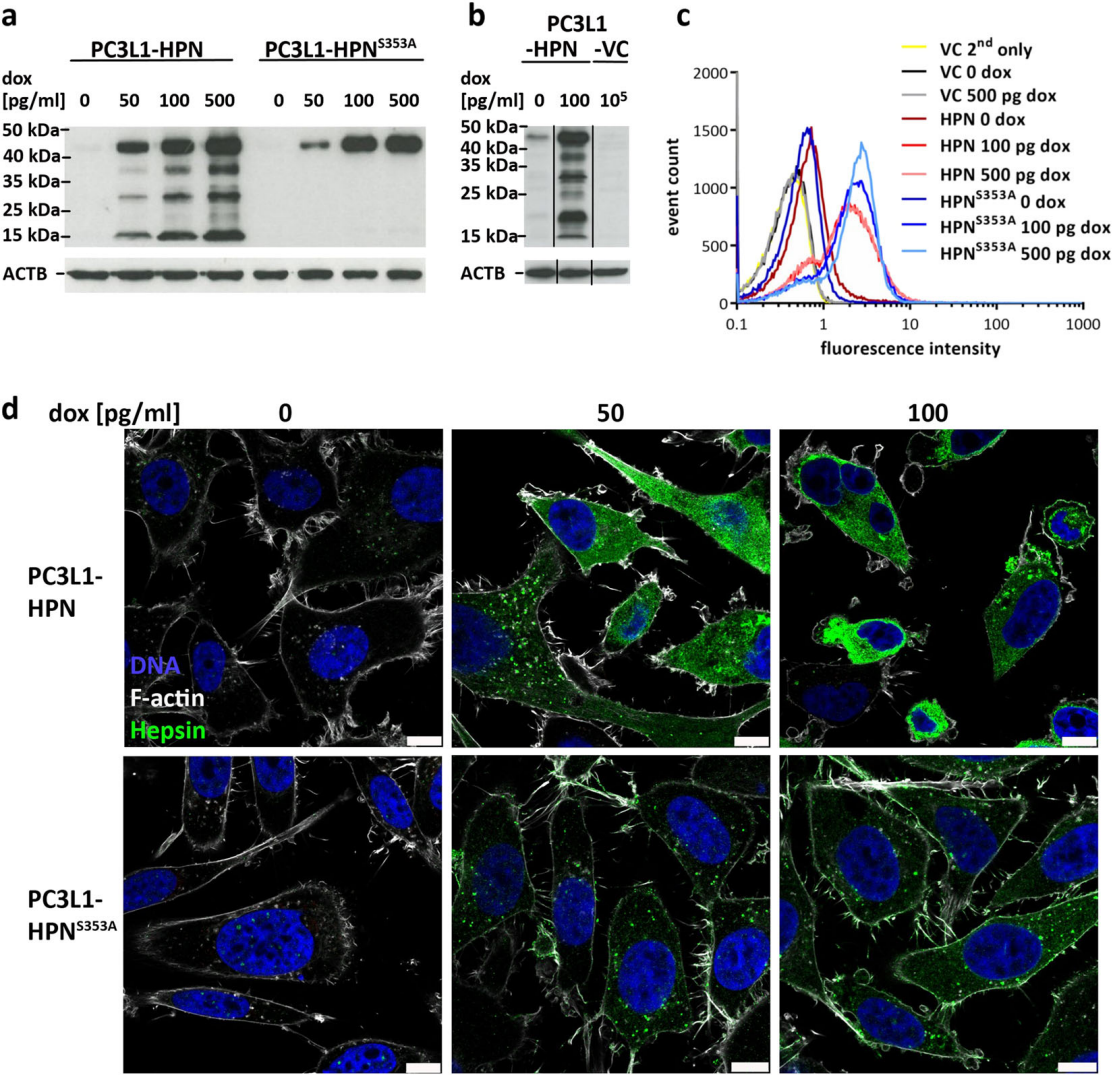
Hepsin expression in isogenic PC-3 cell lines. **a** Western blot analysis of proteolytically active (in PC3L1-HPN) vs. inactive (in PC3L1-HPN^S353A^) hepsin at different concentrations of dox. Sizes of the molecular weight marker are indicated and allow for identification of pro-enzyme (45 kDa) as well as of the protease- (28 kDa) and SRCR-domain- (17 kDa) containing fragments. β-actin (ACTB, lower panel) was used as a loading control. **b** Long term exposure of total protein samples in western blot analysis demonstrates weak expression of the hepsin transgene in the absence of the inductor doxycycline (dox), which is caused by “leakiness” of the construct. Hepsin protein expression is strongly enhanced in the presence of dox, whereas it is absent in dox-induced empty vector control cells (PC3L1-VC). **c** Comparative analysis of hepsin cell surface expression of the isogenic clones PC3L1-HPN, PC3L1-HPN^S353A^, and PC3L1-VC via flow cytometry of live cells. Cell populations are color coded as indicated in the legend. VC 2nd only denotes PC3L1-VC cells which were incubated with the fluorophore coupled secondary antibody only, omitting the antigen-specific primary antibody. **d** Subcellular localization of wild-type (upper panel) vs. mutant (lower panel) hepsin protein at different levels of target gene induction. Images were generated using confocal laser scanning fluorescence microscopy and show representative cell populations (scale bar: 10 µm).

Western blot analysis revealed slightly different expression dynamics, but similar maximum levels of the transgene-encoded proteins at 500 pg/ml dox. Whereas proteolytic processing was not detectable for the mutant enzyme, cleavage products of the wild-type protein at 17 and 28 kDa indicate that the autocatalytic activation described by others^25,26^ is also a major mechanism of hepsin zymogen activation in PC-3 cells (Fig. 1a).

In the uninduced state, flow cytometry revealed slightly stronger surface fluorescence in PC3L1-HPN and PC3L1-HPN^S353A^ when compared with the isogenic empty vector control cell line PC3L1-VC, indicating leaky expression, which was verified for PC3L1-HPN by long term exposure of western blot samples (Fig. 1b, c). Maximum surface expression of both proteins was detected at 100 pg/ml dox, respectively, indicating saturation of membrane localization and distribution of additional protein to other subcellular destinations (Fig. 1c). Microscopy revealed different subcellular localization patterns of wild-type vs. mutant hepsin. In the uninduced state, both proteins revealed a weak and punctate localization. Moderate gene induction at 50 pg/ml dox resulted in partial redistribution of wild-type hepsin to the cytoplasm, whereas mutant hepsin remained in punctate patterns and in the cytoplasmic membrane. Costaining with early endosome antigen 1 (EEA1) revealed partial colocalization of both variants (Supplementary Fig. 1). At 100 pg/ml dox, the punctate staining pattern of wild-type hepsin predominantly changed to homogeneous cytoplasmic staining, whereas the mutant protein retained its original localization (Fig. 1d).

### Adverse effects require hepsin proteolytic activity

**Fig. 2:**
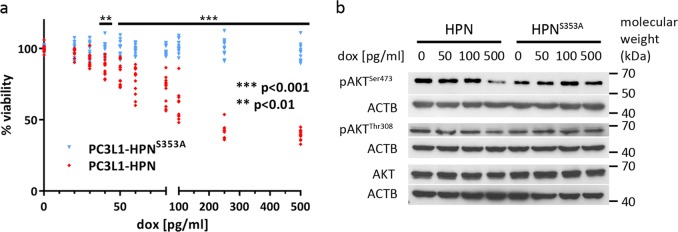
Viability and AKT phosphorylation in isogenic PC3L1-HPN vs. PC3L1-HPN^S353A^ cell populations at different levels of transgene induction. **a** Cell populations were treated with dox as indicated and grown for 72 h. The graph shows mean viability ± standard deviation (SD) as percentages of the untreated control, respectively. Four independent experiments were performed in triplicates (*n* = 12). Statistical significance was calculated using unpaired *t*-test and the Holm–Sidak method for correction for multiple comparisons. Adjusted *p*-values **p < 0.01; ***p < 0.001. Cell population samples are color coded as indicated in the legend. **b** Western blot analysis for expression and phosphorylation of the AKT-protein in cell populations treated with dox for 48 h as indicated. ACTB was probed as a control for equal protein loading.

We previously showed that hepsin-mediated adverse effects could not be reverted by addition of the broad band serine protease inhibitor AEBSF^19^. To investigate whether this was caused by incomplete enzyme inhibition, we compared the isogenic transfectants in side-by-side analyses. Cell adhesion (not shown), viability and PKB/AKT phosphorylation were not affected during overexpression of protease-deficient hepsin (Fig. 2), demonstrating that proteolytic activity is causative for the adverse effects during hepsin overexpression.

### Activation of uPA is not enhanced in response to hepsin overexpression

**Fig. 3:**
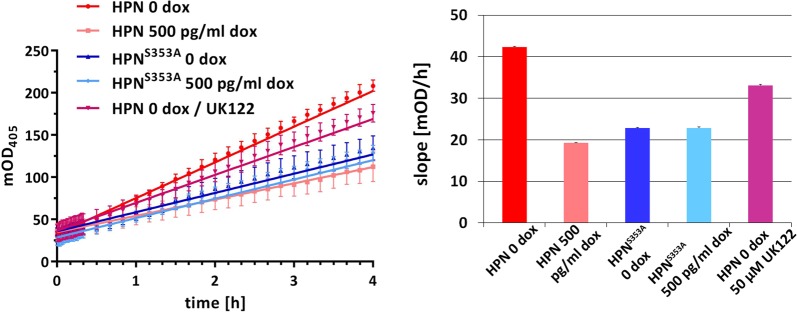
Comparison of uPA activity in PC3L1-HPN and isogenic PC3L1-HPN^S353A^ cell populations in the absence vs. presence of dox. Left diagram: the blank-corrected median intensity values reporting cleavage of an uPA-specific chromogenic substrate were recorded during a 4 h incubation. Linear regression curves and standard errors were determined using four technical replicates, respectively (*n* = 4). UK122 (50 µM) was added 24 h prior to the measurement of uPA activity. The right diagram shows the corresponding slopes of the linear regression curves. The figure shows exemplary data for one out of two experiments with similar results. Cell populations were color coded as indicated in the legend.

When proteolytically processed and bound to its receptor, the serine protease uPA is a potent activator of various pericellular proteases, which itself regulate cell migration, basement membrane remodeling, and invasive (tumor) growth^27^. Hepsin has been described as an activator of pro-uPA^7,28^ and could therefore act as an upstream initiator of metastasis-promoting pericellular proteolytic cascades. Surprisingly, comparative uPA activity assays revealed highest levels for PC3L1-HPN in the absence of dox (Fig. 3), probably resulting from leaky hepsin expression. In the presence of dox, reduced uPA activity correlated with the suppression of viability by ~50% in PC3L1-HPN (Fig. 2a), suggesting that uPA activation per cell is not substantially altered during hepsin overexpression. As expected, expression levels of mutant hepsin did not affect uPA activity levels. The uPA inhibitor UK122^24^ induced a signal reduction in the kinetic analysis (Fig. 3), but did not reverse the loss of viability during hepsin overexpression (Supplementary Fig. 2). Our results suggest that hepsin attained its maximum uPA activation capacity already in the absence of dox, and that hepsin-mediated adverse effects were neither mediated by uPA itself nor by uPA-activated pericellular proteolytic cascades.

### Excess hepsin activity limits c-met maturation, NOTCH activity, and reduces integrin expression

**Fig. 4:**
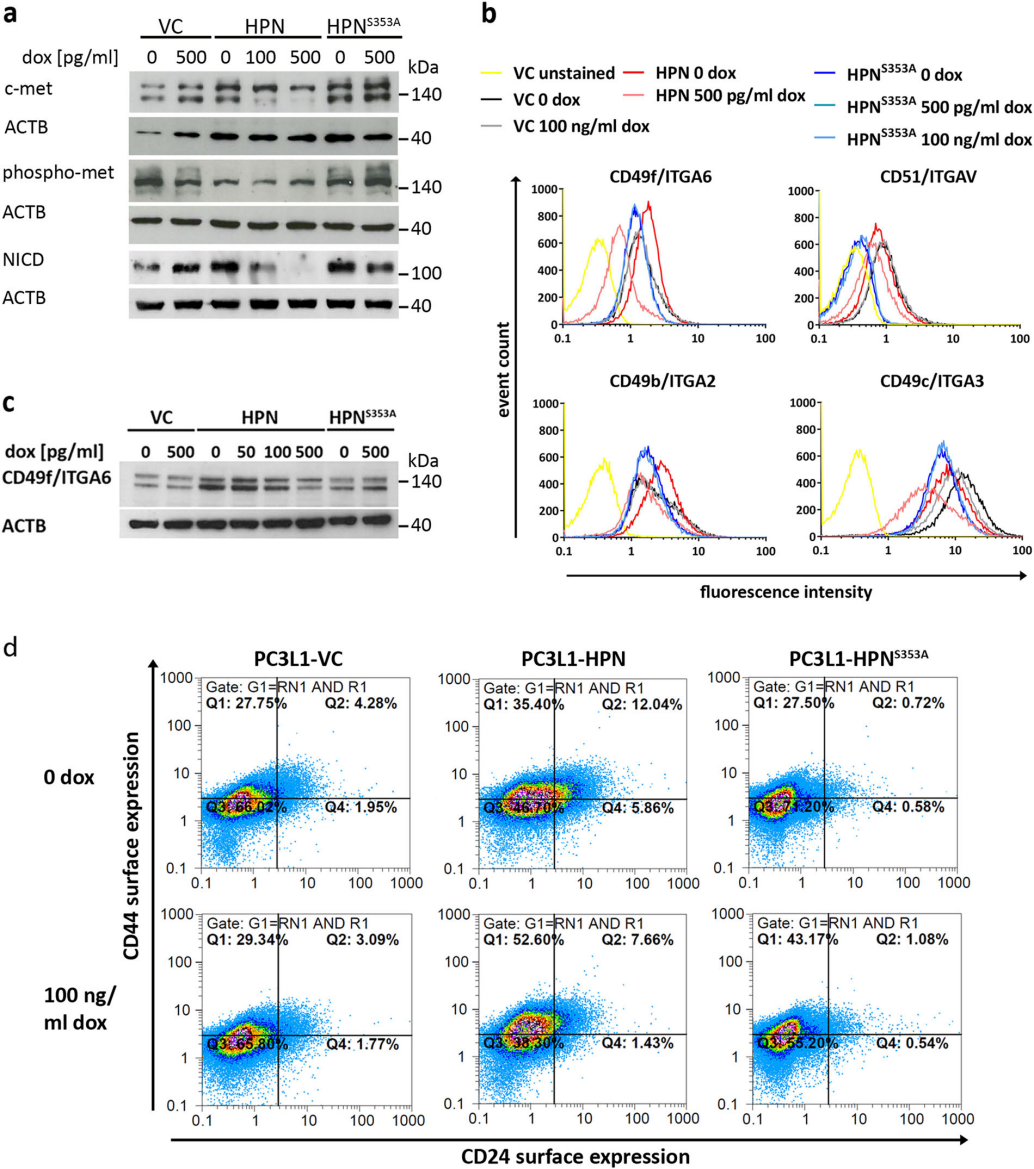
Analysis of HGF/met signaling and stemness/adhesion-associated markers. **a** Western blot analyses subsequent to growth of the indicated cell populations in the absence of FCS for 24 h. The c-met antibody detects the unprocessed single chain precursor protein (upper band, 170 kDa) as well as the mature 140 kDa β-chain (lower band). The phospho-met antibody detects phosphorylated Tyr1234/Tyr1235 residues in the c-met β-chain. ACTB was probed as a control for equal protein loading, respectively. **b** Cell surface integrin profiling at 72 h post target gene induction using flow cytometry of live cells. Representative histogram plots for one of two experimental series showing similar results are depicted. Cell populations are color coded as indicated in the legend. “VC unstained” denotes PC3L1-VC cells which were incubated with the fluorophore coupled secondary antibody only, omitting the antigen-specific primary antibody. **c** Exemplary western blot analysis of CD49f/ITGA6 resulted in the detection of glycosylation variants at ~125 and 150 kDa. ACTB was probed as a control for equal protein loading. **d** Representative two parameter dot plots showing expression levels of CD24 (horizontal axis) vs. CD44 (vertical axis) in double-staining experiments. Cell populations were gated for live cells and subjected to quadrant analysis using identical settings. One of two experimental series providing similar results is shown.

The HGF/c-met pathway was recently identified to induce a stem-like expression signature (increase of CD49b/ITGA2, CD49f/ITGA6, reduction of CD24, activation of Notch signaling) in PCa^29^. Pro-HGF has been described as a hepsin substrate^26,30^. However, as the HGF-receptor c-met is constitutively active in PC-3^31^, HGF activity should be dispensable for c-met signaling in this cell line. Accordingly, no enhancement of c-met phosphorylation, but a decrease of the c-met beta chain (indicating reduced maturation) was detected during hepsin overexpression. Under these conditions, proteolytic cleavage of Notch1 and release of the notch intracellular domain (NICD) was also strongly decreased (Fig. 4a).

Flow cytometry revealed substantially reduced cell surface expression of the stemness-associated integrins α2 (ITGA2/CD49b), and α6 (ITGA6/CD49f), as well as of integrin α3 (ITGA3/CD49c) during overexpression of wild-type hepsin, while integrin αV (ITGAV/CD51) expression remained largely constant (Fig. 4b). Interestingly, neither the cell-impermeable inhibitor AEBSF nor the uPA inhibitor UK122 could rescue the loss of ITGA6/CD49f at the cell surface, indicating that reduced surface expression may not be mediated by pericellular proteolysis through serine proteases (Supplementary Fig. 3). Induction of PC3L1-HPN^S353A^ or the vector control cell line PC3L1-VC did not significantly affect surface marker expression. CD49b/ITGA2 and CD49f/ITGA6 were slightly stronger expressed in cells expressing basal levels of wild type compared with protease-deficient hepsin, which was exemplarily verified by western blot analysis for CD49f/ITGA6 (Fig. 4c). CD24, a surface marker of neuroendocrine carcinomas^32,33^ with negative predictive value in PCa^34^ revealed enhanced cell surface expression in presence of basal levels of wild-type hepsin, which returned to lower values during overexpression (Fig. 4d). Surface expression of CD44 (Fig. 4d) and CD151 (Supplementary Fig. 4) was not consistently altered. In summary, our data indicate stem-like marker protein expression in PC-3 during low (uninduced) expression of wild-type hepsin, which is HGF-independent and partially lost in response to overexpression.

### Hepsin overexpression increases acidic vesicle content and autophagic flux in vitro

**Fig. 5:**
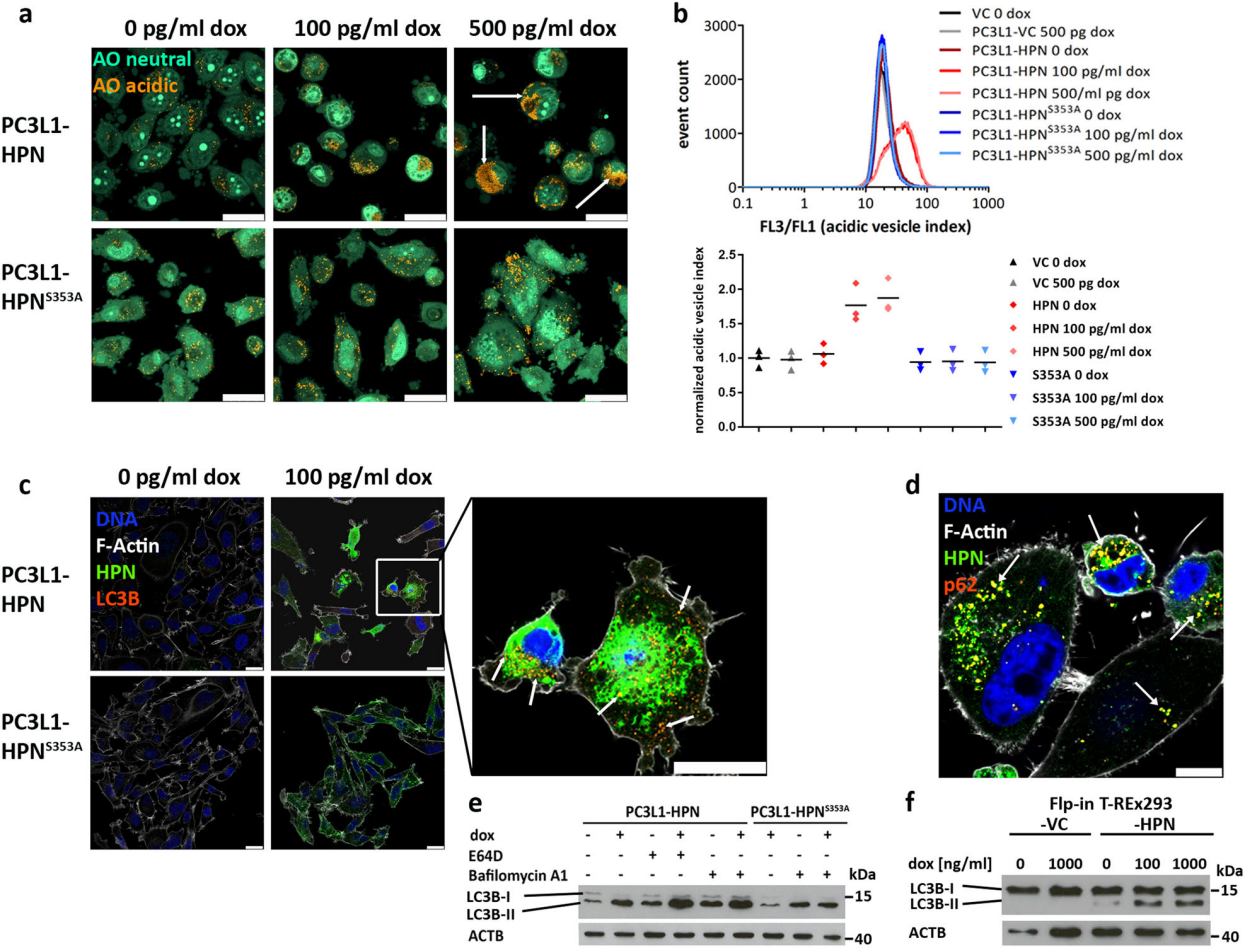
Excess hepsin activity increases autophagic flux. **a** Confocal Laser Scanning microscopy of PC3L1-HPN and PC3L1-HPN^S353A^ cells stained with acridine Orange (AO) 72 h post transgene induction using dox concentrations as indicated. Massive accumulation of acidic vesicles (AO acidic) in dox-treated PC3L1-HPN is exemplarily indicated by arrows (scale bar: 25 µm). **b** Quantitative analysis of acidic vesicle accumulation (acidic vesicle index) in PC3L1-VC, PC3L1-HPN, and PC3L1-HPN^S353A^ by flow cytometry 72 h post addition of dox. The exemplary histogram plot indicates the ratio of orange vs. green fluorescence intensity per cell in one experimental series. The lower graph indicates the mean ratios based on averaged mean fluorescence intensities of three independent experimental series (*n* = 3). The mean value for untreated PC3L1-VC populations was set to one and the values for other populations were adjusted accordingly. Cell populations are color coded as indicated in the legend. **c** Immunofluorescence analysis of hepsin and LC3B expression 48 h post target gene induction using dox concentrations as indicated (scale bar: 25 µm). Dox-treated PC3L1-HPN revealed expression of LC3B punctae, which partially colocalize with hepsin, as indicated by yellow color and exemplarily marked by arrows in the magnified image section. **d** Immunofluorescence staining demonstrates colocalization of hepsin and p62/SQSTM1, as indicated by yellow color and exemplarily marked by arrows in PC3L1-HPN cells in the presence of 100 pg/ml dox (scale bar: 10 µm). **e** Western blot analysis of LC3B expression (upper band: LC3B-I, lower band: LC3B-II) in PC3L1-HPN and -HPN^S353A^ cell populations treated with, E64D (10 µM, 24 h) and/or Bafilomycin A1 (100 nM, 4 h) in absence or presence (100 pg/ml, 48 h) of dox as indicated. ACTB was probed as a control for equal protein loading. **f** Western blot analysis of LC3B expression (upper band: LC3B-I, lower band: LC3B-II) in Flp-in T-Rex293-VC and -HPN cell populations in the absence or presence of dox (48 h) as indicated. ACTB was probed as a control for equal protein loading.

Since loss of viability is accompanied by redistribution of hepsin from endosomal compartments to the cytoplasm during overexpression, we initially hypothesized LMP as a possible cause for the phenomena observed. To evaluate lysosome content and integrity, we performed live cell fluorescence microscopy using AO. Induction of wild-type hepsin did not reduce acidic compartment-associated orange emission of AO, demonstrating that excess hepsin activity does not correlate with LMP (Fig. 5a). In contrast, quantitation of orange vs. green staining by flow cytometry revealed an increase in acidic vesicle content during overexpression of wild-type hepsin (Fig. 5b), which could be indicative for increased autophagy^35^. Immunofluorescence analysis revealed LC3B-II punctae, which indicate presence of autophagosomes, in several cells overexpressing wild-type hepsin, but not in uninduced PC3L1-HPN and in PC3L1-HPN^S353A^ cell populations (Supplementary Fig. 5). LC3B-II punctae partially colocalized with the hepsin signal in double stainings, suggesting presence of hepsin in autophagosomes (Fig. 5c and Supplementary Fig. 6). SQSTM1/p62, an adaptor protein guiding polyubiquitinylated cargo to autophagosomes, was found to co-localize with both wild-type (Fig. 5d) and protease-deficient hepsin (Supplementary Fig. 7a), however, its expression did not correlate with transgene expression (Supplementary Fig. 7b). Autophagosome abundance may either result from increased autophagic flux or from a deficiency in autophagosome clearance, i.e., their fusion with lysosomes^36^. To check whether autophagic flux is accelerated, we applied LC3B immunoblotting in the absence vs. presence of inhibitors which interfere with autophagolysosome homeostasis. Treatment with both bafilomycin A1, an inhibitor of the vacuolar V-type H^+^-ATPase (inhibiting lysosome acidification), and with E64d, an inhibitor of lysosomal proteases, demonstrates successful blockade of autophagosome cargo clearance by increasing LC3B-II (Fig. 5e and Supplementary Fig. 8). Thus, an acceleration of autophagic flux appears to be causal for the increase of LC3B-II in hepsin-overexpressing PC3L1-HPN. To investigate whether hepsin-induced autophagy functions in a cytoprotective manner, cell viability was analyzed in presence of the autophagy inhibitors 3-methyladenine and chloroquine. No further decrease in cell viability could be detected in the presence of the inhibitors during overexpression of wild-type hepsin (Supplementary Fig. 9), indicating that the autophagic response is not of cytoprotective nature.

To assess whether increased autophagy is restricted to the PC-3 cell model or a general phenomenon following hepsin overexpression, we investigated LC3B-II abundance (i) by western blot analysis in stably transfected human embryonal kidney HEK293 cells providing inducible overexpression of wild-type hepsin, and (ii) by double immunofluorescence analysis (HPN/LC3B) in androgen-receptor (AR)-positive LNCaP PCa cells post transient overexpression of wild-type vs. protease-deficient hepsin. Similar to PC-3, hepsin induction increased LC3B-II expression in HEK293 cells (Fig. 5f). In LNCaP cells, strong staining for hepsin was present in ~20% of the cell population, thereby reflecting the average transfection efficiency as determined using a GFP expression plasmid (not shown). The localization patterns of wild-type and protease-deficient hepsin were reminiscient to those observed in PC-3 cells, respectively. Wild-type hepsin showed strong cytoplasmic staining and partial colocalization with the LC3B signal, whereas cells transiently expressing protease-deficient hepsin were negative for LC3B (Supplementary Fig. 10). These results demonstrate that autophagy is a common cellular response to excess hepsin proteolytic activity.

### Hepsin overexpression increases LC3B punctae frequency in an in vivo xenograft model

**Fig. 6:**
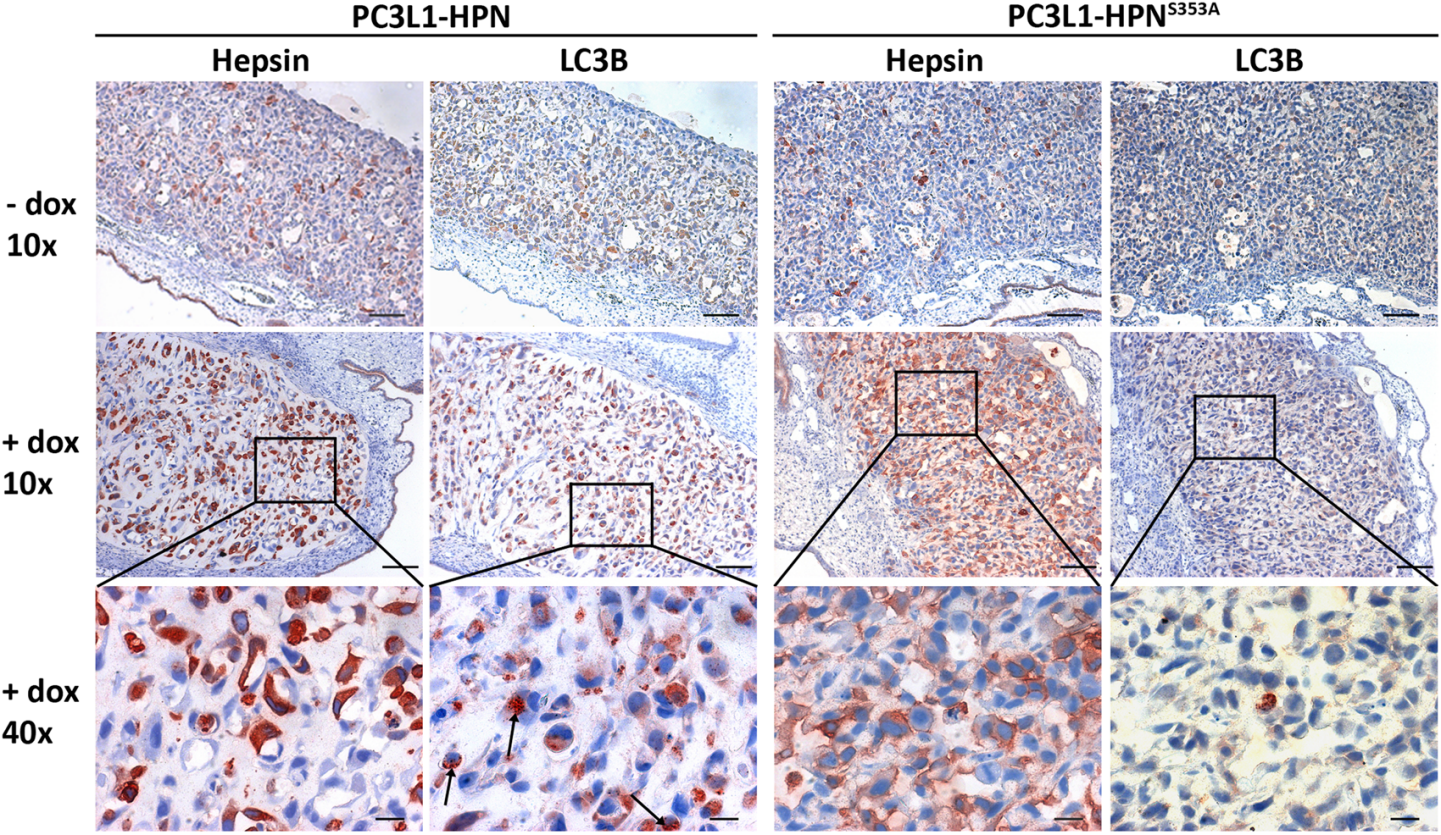
Overexpression of wild-type hepsin induces cytoplasmic localization of the enzyme and autophagy in CAM xenografts. PC3L1-HPN and PC3L1-HPN^S353A^ cells were transplanted on the CAM and grown in the absence and presence of dox (100 ng/ml, topical application for 72 h). Exemplary micrographs show the expression of hepsin and LC3B in the indicated tumors at tenfold magnification (scale bar: 100 µm) and 40-fold magnification (scale bar: 20 µm). Note the cytoplasmic localization of wild-type vs. membraneous localization of proteolytically inactive hepsin. Cells containing LC3B punctae are exemplary indicated by arrows.

We recently demonstrated invasive growth of PC3L1-HPN cells in the CAM xenograft model^19^. Tumors were phenotypically different in the presence of dox, showing a significantly lower cell content and mitotic index, and increased apoptosis. To check whether this phenotype coincides with an increase of autophagy, we investigated serial sections of PC3L1-HPN vs. PC3L1-HPN^S353A^-derived tumors in the presence and absence of dox for expression of hepsin and LC3B via immunohistochemistry. Upon overexpression, intracellular localization of wild-type hepsin was primarily cytoplasmic, whereas protease-deficient hepsin was mainly found in the cytoplasmic membrane, thus resembling the intracellular distribution found in vitro (see Fig. 1d). We found a strong increase of LC3B punctae in tumors overexpressing wild type, but not in those overexpressing protease-deficient hepsin, indicating that excess activity correlates with cytoplasmic localization of the enzyme and induces autophagy in this in vivo xenograft model (Fig. 6).

### Hepsin overexpression induces the unfolded protein response

**Fig. 7:**
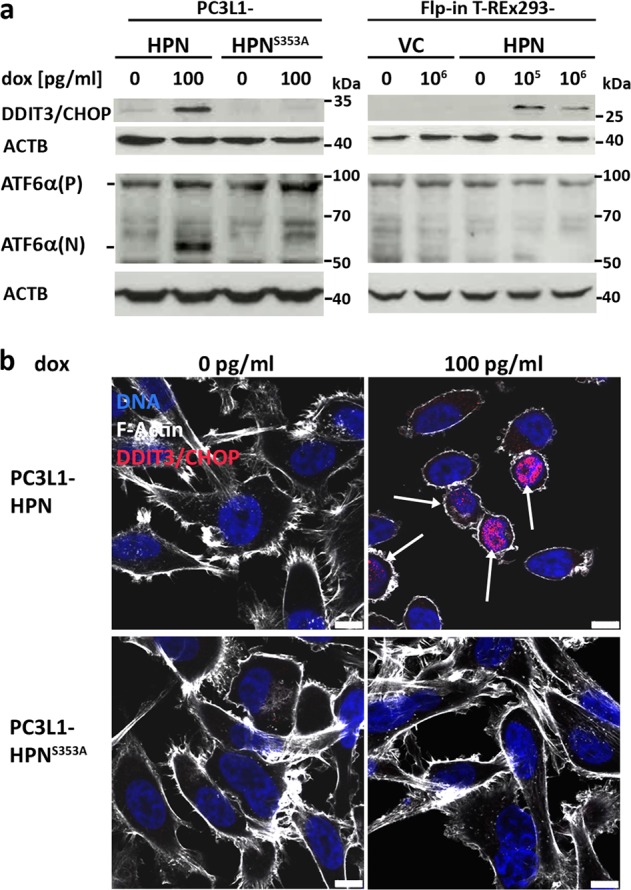
Excess hepsin activity induces proteolytic cleavage of ATF6α and expression and nuclear localization of CHOP/DDIT3. **a** Western blot analysis for expression of DDIT3/CHOP (upper panel) and expression/cleavage of ATF6α (lower panel, ATF6α(P) denotes the ~95 kDa precursor protein, ATF6α(N) denotes the ~60 kDa transcription factor) in PC3L1- and Flp-in T-REx-derived cell populations treated with dox for 72 h as indicated. ACTB was probed as a control for equal protein loading. **b** Immunofluorescence analysis for expression and localization of the DDIT3/CHOP protein in PC3L1-derived cell populations treated with dox for 72 h as indicated (scale bar: 10 µm).

An increase in secretory protein synthesis may cause ER stress, which activates the unfolded protein response (UPR), followed by ER-associated degradation (ERAD) via either the ubiquitin-proteasome system (ERAD type I) or in an autophagy-lysosome-dependent manner (ERAD type II)^37^. Western blot analysis demonstrated a strong induction of the UPR key transcription factor CHOP in response to overexpression of wild type, but not protease-deficient hepsin in PC-3 cells, as well as in HEK293 cells overexpressing hepsin. In PC-3 but not in HEK293 cells, this was accompanied by cleavage of the ER stress sensor activating transcription factor 6 alpha (ATF6α) and formation of the ATF6α(N) fragment, which transcriptionally activates UPR proteins, among them CHOP^38–40^ (Fig. 7a). Immunofluorescence analyses revealed nuclear translocation of CHOP in presence of wild-type, but not protease-deficient hepsin in PC-3 (Fig. 7b) as well as in transiently transfected AR-positive LNCaP cells (Supplementary Fig. 10), thereby indicating that induction of the UPR is a general response to excess hepsin proteolytic activity. However, differential activation of ATF6 indicates that ER stress sensing pathways activated in response to excess hepsin activity may be tissue/cell type specific.

### Inhibitors of ER translocation, ER stress, and protein degradation differentially affect viability during excess hepsin activity

**Fig. 8:**
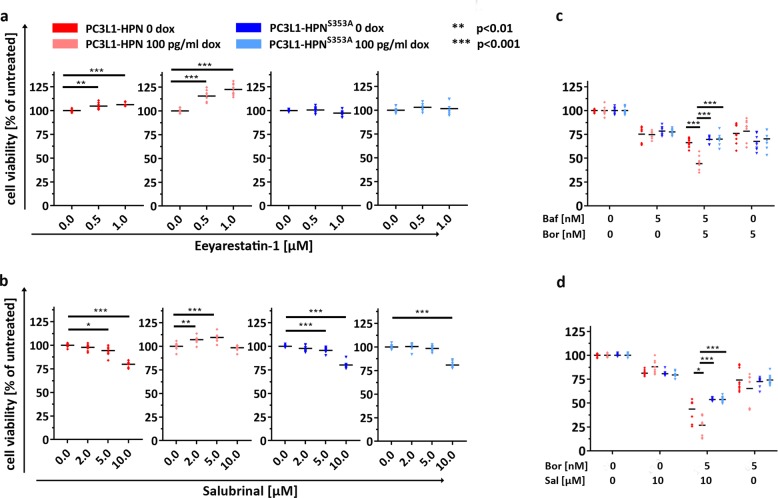
Inhibitors of ER translocation, ER stress, and protein degradation differentially affect viability during excess hepsin activity. Viability of cell populations in response to 48 h incubation with different concentrations of Eeyarestatin-1 (**a**), salubrinal (Sal) (**b**), bafilomycin A1 (Baf)/bortezomib (Bor) (**c**), and bortezomib/salubrinal as indicated. Graphs represent the mean viability ± standard deviation as percentages of the untreated control cell population, respectively. Please note that the reduction of viability in response to overexpression of wild-type hepsin is not apparent in this mode of presentation, but was similar as depicted in Fig. 2a. Three independent experiments were conducted in triplicates (*n* = 9), respectively.

The sorting of proteins to the secretory pathway occurs at the rough ER and is mediated by a complex containing the sec61 protein^41^. As hepsin attains its proteolytic activity during the passage through the secretory pathway, we speculated that the inhibition of sec61-mediated protein translocation may suppress hepsin-associated adverse effects. To check this hypothesis, we applied the translocation inhibitor eeyarestatin-1^42^ and measured the viability in presence (100 pg/ml) vs. absence of dox. Continuous low dose exposition to eeyarestatin-1 induced a slight but significant increase of viability in uninduced PC3L1-HPN, which was further enhanced during hepsin overexpression. In contrast, eeyarestatin-1 did not affect viability in PC3L1 expressing protease-deficient hepsin, indicating that the suppression of hepsin maturation promotes viability in PC3L1-HPN (Fig. 8a). Salubrinal, which indirectly reduces protein translocation to the ER by inhibiting dephosphorylation of the eukaryotic translation initiation factor 2 subunit alpha^43^, similarly increases viability at low doses (up to 5 µM) during overexpression of wild-type, but not protease-deficient hepsin (Fig. 8b). However, salubrinal did not reduce nuclear presence of CHOP during hepsin overexpression, and western blot analysis revealed a slight increase of LC3B-II in response to salubrinal treatment in all PC-3 cell populations, irrespective of expression and proteolytic activity of the transgene (Supplementary Fig. 11).

The inhibition of protein degradation pathways during ER stress is regarded as a promising option for cancer therapy^44^. Since excess hepsin activity induces ER stress, we assumed that inhibition of autophagy (by bafilomycin A1) and/or the ubiquitin-proteasome pathway (by bortezomib) may be particularly efficient for cell killing during overexpression of wild-type hepsin. Whereas the suppression of viability at single inhibitor treatment was not more pronounced during excess hepsin activity, simultaneous administration of both inhibitors revealed a significantly increased growth-inhibitory effect (Fig. 8c). Surprisingly, a similar effect could be achieved by combined administration of bortezomib and salubrinal (Fig. 8d), indicating that the suppression of ERAD pathways, combined with modulation of ER stress, may be a therapeutic option for hepsin-overexpressing PCa. Apoptosis, which can be induced by CHOP activity, does not seem to be predominant in hepsin-mediated effects, as determined by annexin V staining in absence vs. presence of inhibitor (combinations). We detected an overall slightly higher percentage of apoptotic cells during hepsin overexpression, which correlates with recent in vivo observations^2,19^ and was not considerably altered by the inhibitors (Supplementary Fig. 12).

## Discussion

Due to its overexpression, extracellular proteolytic activity, and functional involvement in basement membrane degradation and invasive tumor growth, hepsin was declared as a promising and druggable cancer target^1,2,45–47^. However, attempts to correlate mRNA and protein expression levels with staging/grading yielded controversial results for PCa^1,14^ and breast cancer^15,46^. The hepsin paradox describes reduced expression of hepsin in advanced PCa and other solid tumors and absence in metastasis-derived cancer cell lines, as well as adverse effects during transgenic overexpression^18^. The mechanistic basis for this phenomenon is yet unresolved. Our present study sheds light on the nature of the hepsin paradox, aids to interpret clinical observations, and suggests the interference with ER stress and protein degradation pathways as candidate adjuvant therapy for hepsin-overexpressing tumors.

We could demonstrate that excess hepsin proteolytic activity is a prerequisite for the adverse effects associated with hepsin overexpression. Comparatively weak expression levels of hepsin seem sufficient for the execution of at least some of its cancer-promoting effects, favoring oncogenic signaling, and stemness in PC3L1-HPN cells. At higher expression levels, excess hepsin activity becomes a burden for the cancer cell. Here, hepsin is targeted to intracellular degradation, as indicated by colocalization with degradation-associated proteins such as p62/SQSTM1 and LC3B^48^. Consequently, both inhibition of sec61-mediated transport between cytoplasm and ER, as well as a general decrease in protein translation increased viability during hepsin overexpression. ER stress and enhanced autophagy in response to hepsin overexpression were not limited to the PC-3 cell line with all its cancer-specific peculiarities, but also occurred in AR-positive LNCaP PCa and in HEK293 cells, which demonstrates generalizability of our findings. Cytoplasmic localization of hepsin was not only observed in PC-3 and LNCaP cells but also in other experimental systems, as well as in patient samples. Miao et al. reported a switch from desmosome-associated to cytoplasmic localization of hepsin in experimental ovarian cancer, which was dependent on proteolytic activity of the enzyme^5^. During experimental mammary carcinogenesis in vitro, Partanen et al.^6^ demonstrated a switch from desmosome-associated to cytoplasmic localization of hepsin in response to loss of the tumor suppressor LKB1. Further functional studies revealed insights into mechanistic details of hepsin-induced disruption of epithelial cohesion in vitro, which includes degradation of its inhibitor HAI-1, increased pericellular proteolysis, induction of met activity, as well as the proteolysis-associated disintegration of desmosomes and hemidesmosomes, which also coincided with a translocation of hepsin from the cytoplasmic membrane to the cytosol^7^. Partanen et al. suggested a role for “liberated” (i.e., cytosolic) hepsin in tumor-promoting proteolytic events^49^. However, our data rather suggest proteolysis of at least some pericellular targets at low/basal expression levels of the enzyme, whereas cytoplasmic localization rather seems to reflect hepsin activity-associated ER stress and proteotoxicity. We propose that the redistribution of hepsin is the result of an ERAD-mediated transport of the protein back into the cytosol, followed by autophagic or proteasome-based degradation. Excess hepsin activity further coincides with activation of the unfolded protein response and a lower surface expression of integrins and c-met, indicating a shutdown of protein maturation in the secretory pathway.

Proteotoxicity in tumors could explain the inverse correlation of hepsin (cytoplasmic) expression levels and patient prognosis, as described by Pelkonen et al. for breast^15^ and Dhanasekaran et al.^14^ for prostate cancer. In a recent breast cancer study, the expression of hepsin was reduced in tumors when compared with adjacent healthy tissue. In vitro, ubiquitination and proteasomal degradation of hepsin occurred in response to cathepsin D expression in breast cancer cells, which led to increased metastasis in an in vivo model^50^. Next to giving interesting insights into molecular interactions of hepsin, this study provides a first mechanistic explanation for the detection of hepsin in the cytosol.

Protein recycling/degradation pathways such as autophagy and the ubiquitin-proteasome pathway are essential tools for the management of chronic ER stress^51^. Particularly cancer cells are frequently challenged by massive production of oncoproteins, which may overcharge the quality control and refolding mechanisms^44^. In line with the proposed mechanism of the adverse effects of wild-type hepsin, monotreatment with eeyarestatin-1 and salubrinal, which both reduce the amount and activity of hepsin in the secretory pathway, led to enhanced viability during overexpression of wild-type hepsin. Recently, the simultaneous suppression of the ubiquitin-proteasome degradation pathway and autophagy was found to enhance cell death in ER-stressed pancreatic cancer cells^52^. In line with this strategy, the combined administration of Bortezomib and Bafilomycin A1 was significantly more efficient in the suppression of cell growth during overexpression of wild-type hepsin in PC-3. Surprisingly, although salubrinal alone was protective for hepsin-overexpressing cells, combined administration with bortezomib also induced significantly stronger suppression of viability when compared with cells lacking hepsin overexpression. Although counter-intuitive, a similar effect was previously observed in multiple myeloma^53^ and leukemic cells^54^ and suggests further salubrinal-mediated effects, which are yet not completely understood in detail. However, as salubrinal exerts protective effects in noncancerous tissues^55,56^, it might be a promising sensitizer for anticancer approaches targeting proteotoxicity.

Hepsin-associated adverse effects in cancer cells are most likely also influenced by tissue-specific factors in a given microenvironment. These may include the ECM provided^19^ as well as presence and availability of endogenous inhibitors such as e.g., HAI-1 and HAI-2, which also interact with other TTSP^7,30^. This complex situation may explain the controversial results achieved in different transgenic models, and it is likely that the threshold expression for hepsin-mediated adverse effects varies depending on these factors. However, whether cytoplasmic localization of hepsin is a universal indicator for ER stress and proteotoxicity in cancer cells, which sensing pathways and downstream phenotypes are preferentially involved, and whether this phenotype affects further relevant parameters such as e.g., tumor immunogenicity, must be investigated in future studies using clinical samples. A more detailed knowledge of these processes could imply a therapeutic strategy including interference with ER stress, UPR, and protein degradation pathways for hepsin-overexpressing tumors.

## Supplementary information

Supplementary Material
