# Correction: Excess hepsin proteolytic activity limits oncogenic signaling and induces ER stress and autophagy in prostate cancer cells

**DOI:** 10.1038/s41419-020-2321-7

**Published:** 2020-02-12

**Authors:** Ramona Willbold, Katharina Wirth, Thomas Martini, Holger Sültmann, Christian Bolenz, Rainer Wittig

**Affiliations:** 1 Biology group, Institute for Laser Technologies in Medicine and Metrology (ILM) at the University of Ulm, Helmholtzstr. 12, 89081 Ulm, Germany; 2 Dept. of Urology, Ulm University Hospital, Albert-Einstein-Allee 23, 89081 Ulm, Germany; 3 Cancer Genome Research, German Cancer Research Centre (DKFZ) and German Cancer Consortium (DKTK), Im Neuenheimer Feld 280, 69120 Heidelberg, Germany

**Keywords:** Oncogenes, Urological cancer

**Correction to: Cell Death and Disease**

10.1038/s41419-019-1830-8 published online 09 August 2019

This Article was originally published without the accompanying Supplementary figures. The error has now been fixed and the Supplementary Information is available to download from the HTML version of the Article.

