# Supplementary material for: Excess hepsin proteolytic activity limits oncogenic signaling and induces ER stress and autophagy in prostate cancer cells
Source: Cell Death Dis. 2019 Aug 9;10(8):601. doi: 10.1038/s41419-019-1830-8 (PMC6689070; doi:10.1038/s41419-019-1830-8)
Supplement: Supplementary file 1 — Supplementary Material [file 41419_2019_1830_MOESM1_ESM.docx]

## Supplementary Information


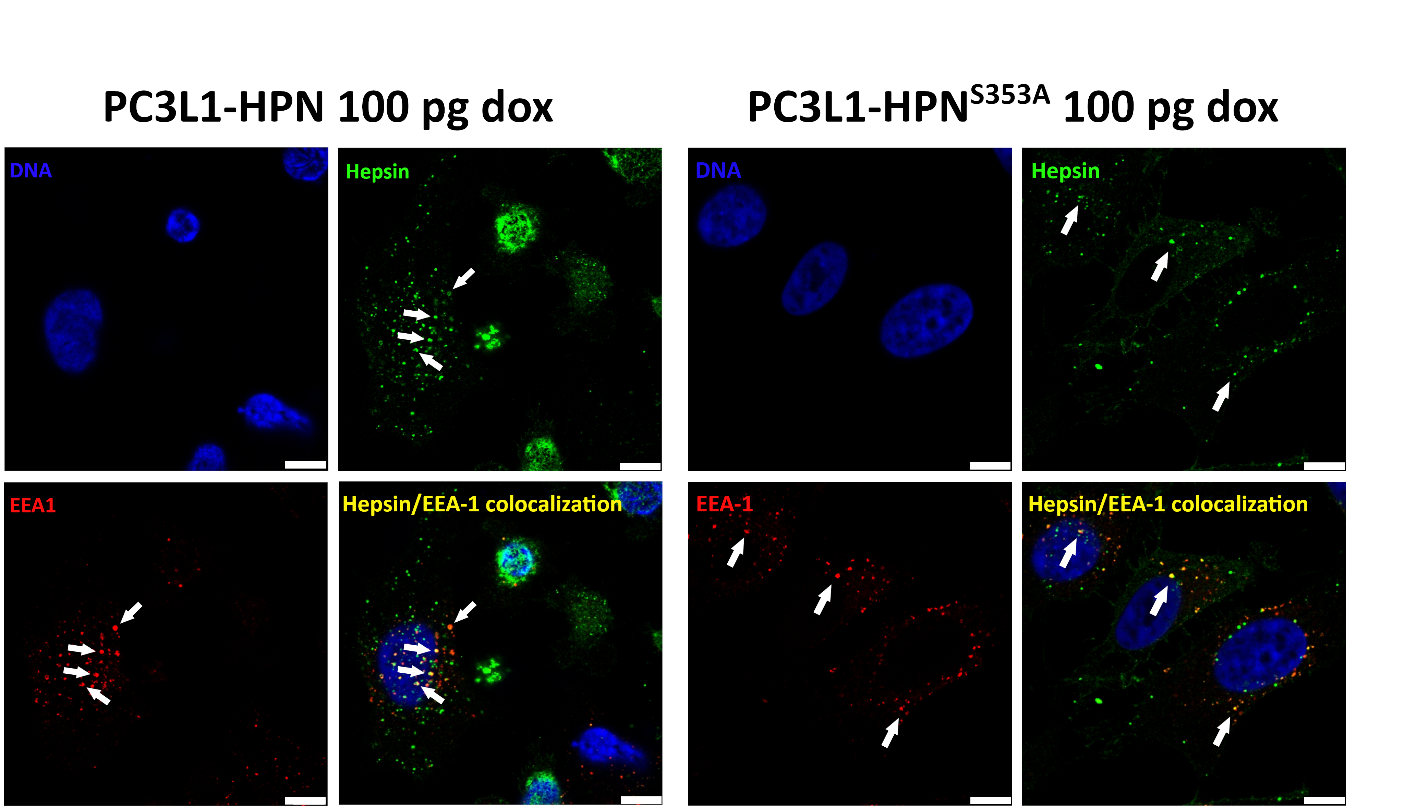


**Supplementary Figure 1:** Co-staining of hepsin and EEA-1 in dox-induced PC3L1-HPN expressing wild type hepsin (left) and PC3L1-HPN^S353A^ expressing protease-deficient hepsin (right). Single channel images and merged images are shown, respectively, and targets are colour-coded as indicated in the images. Co-localization of hepsin and EEA-1 is characterized by yellow colour and exemplarily indicated by arrows (scale bar: 10 µm).


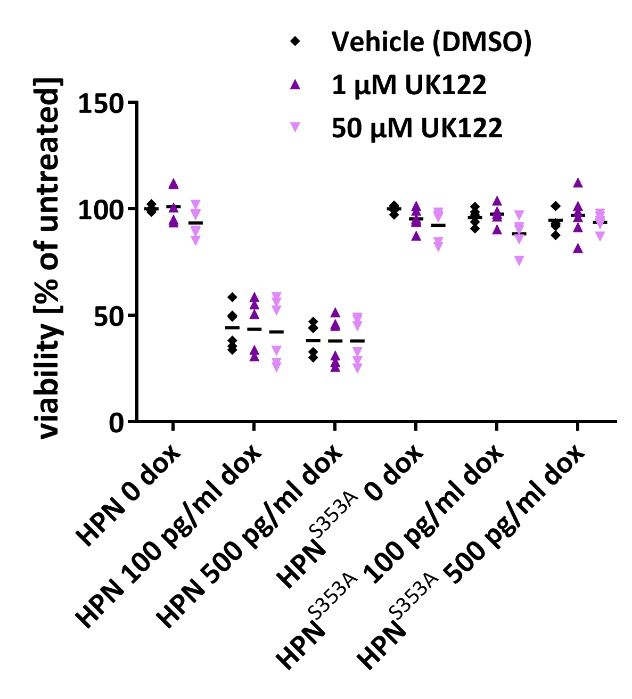


**Supplementary Figure 2:** Viability of PC3L1-HPN and PC3L1-HPN^S353A^ in response to 48 h treatment with u-PA inhibitor UK122 at different concentrations and expression levels of the transgene as indicated. The graph shows mean viability + standard deviation (SD) as percentages of the untreated/uninduced control, respectively. Two independent experiments were performed in triplicates (n=6).


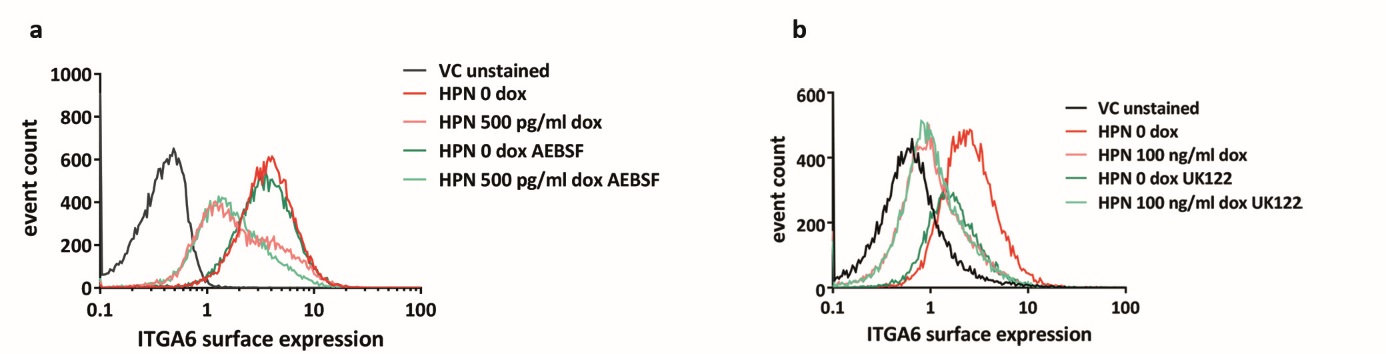


**Supplementary Figure 3:** Cell surface expression analysis of CD49f/ITGA6 antigen in the presence of protease inhibitors. Cell populations are color coded as indicated in the legend. “HPN” denotes PC3L1-HPN cells. “VC unstained” denotes PC3L1-VC cells which were incubated with the fluorophore coupled secondary antibody only, omitting the antigen-specific primary antibody. (a) Twenty-four hours post seeding and hepsin induction, the broadband serine protease inhibitor AEBSF was added to the indicated cell populations at a concentration of 150 µM. (b) Twenty-four hours post seeding and hepsin induction, the small molecule uPA inhibitor UK122 was added to the indicated cell populations at a concentration of 50 µM.


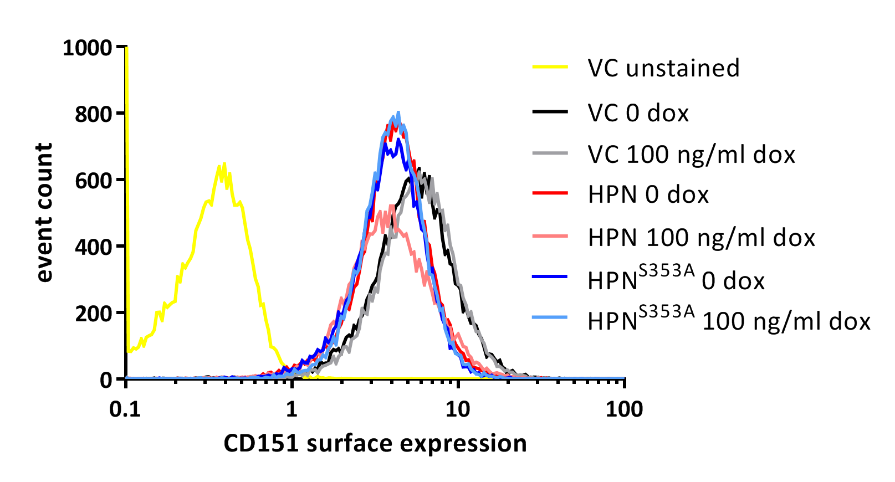


**Supplementary Figure 4:** Cell surface expression analysis of the CD151 antigen at 72 h post target gene induction. The histogram plot shows the results for one of two experimental series with similar outcomes. Cell populations are color coded as indicated in the legend. “VC unstained” denotes PC3L1-VC cells which were incubated with the fluorophore coupled secondary antibody only, omitting the antigen-specific primary antibody.


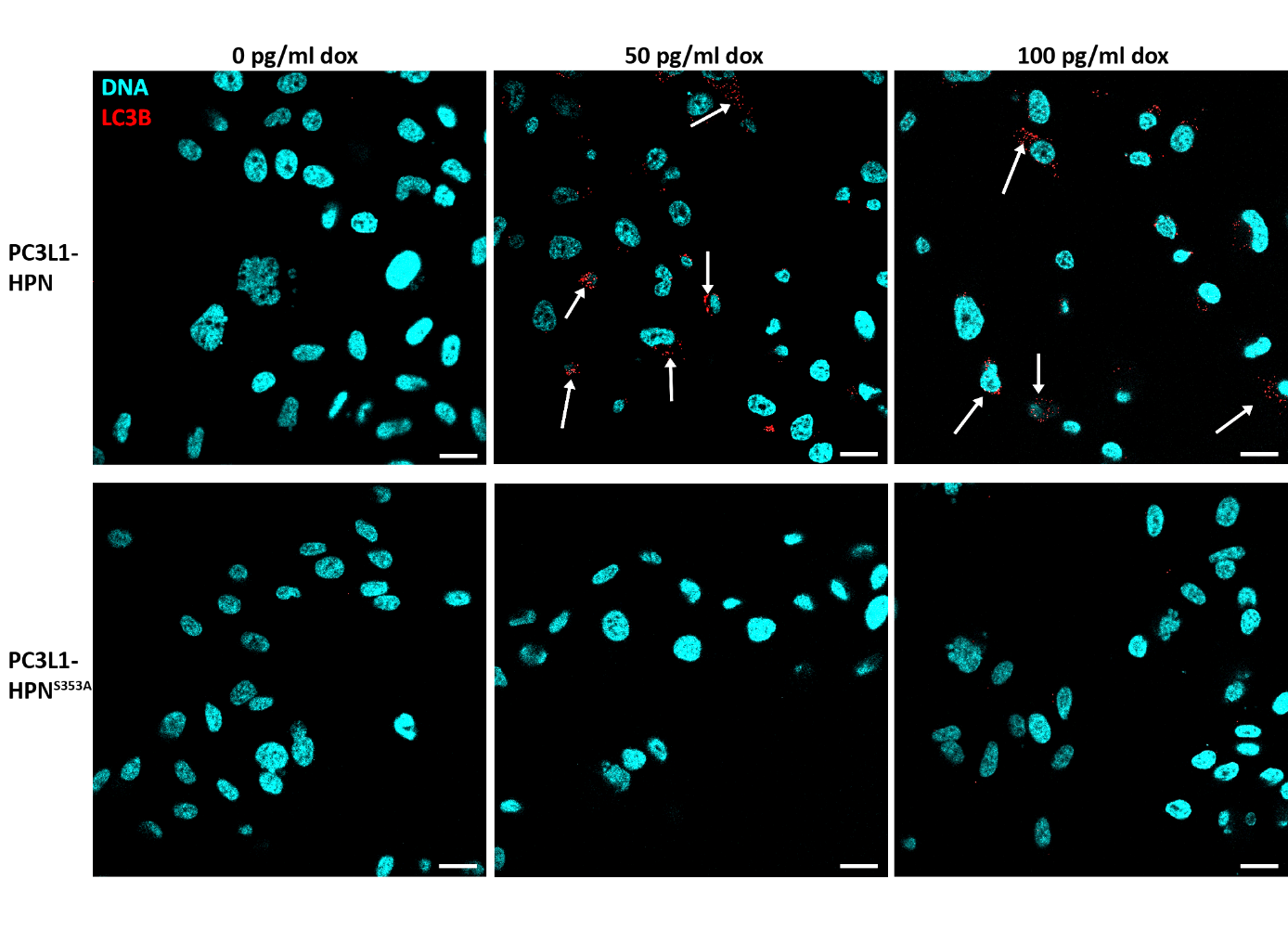


**Supplementary Figure 5:** Increase of LC3B-II punctae in PC3L1-HPN (upper panel) vs. PC3L1-HPN^S353A^ (lower panel) cells at different levels of target gene induction as indicated. Images were generated using confocal laser scanning fluorescence microscopy and show representative cell populations. Cells containing abundant LC3B punctae are frequently present in dox-induced PC3L1-HPN and are exemplarily indicated by arrows. (scale bar: 25 µm).


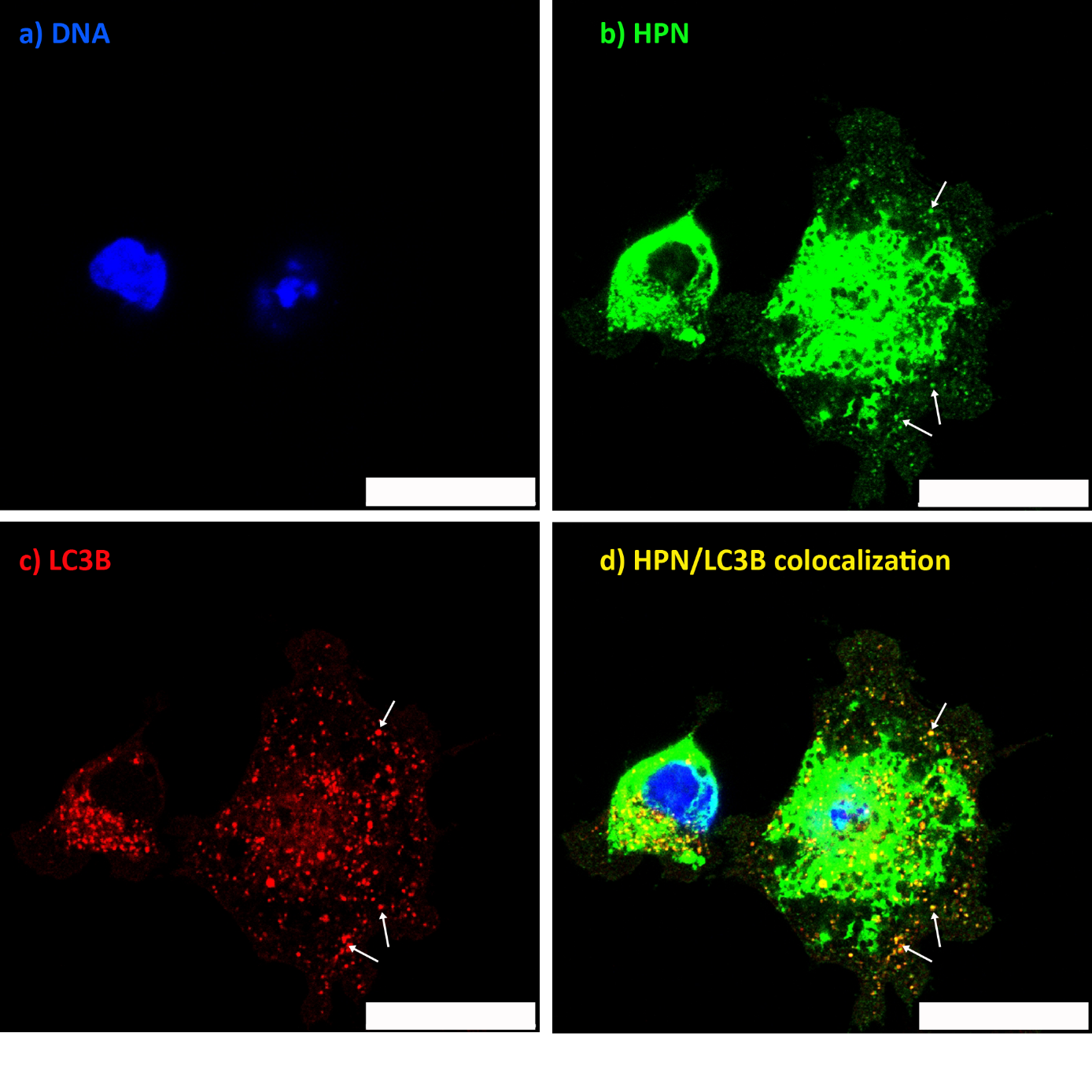


**Supplementary Figure 6:** Co-localization of hepsin and LC3B-II punctae during overexpression of hepsin, as determined by confocal laser scanning fluorescence microscopy. Images a-c show single channel fluorescence for DNA (a), hepsin (HPN, b) and LC3B (c), image d shows and overlay of a-c. Hepsin and LC3B show a partial co-localization, which is indicated by yellow color and exemplarily depicted by arrows (scale bar: 25 µm).

##
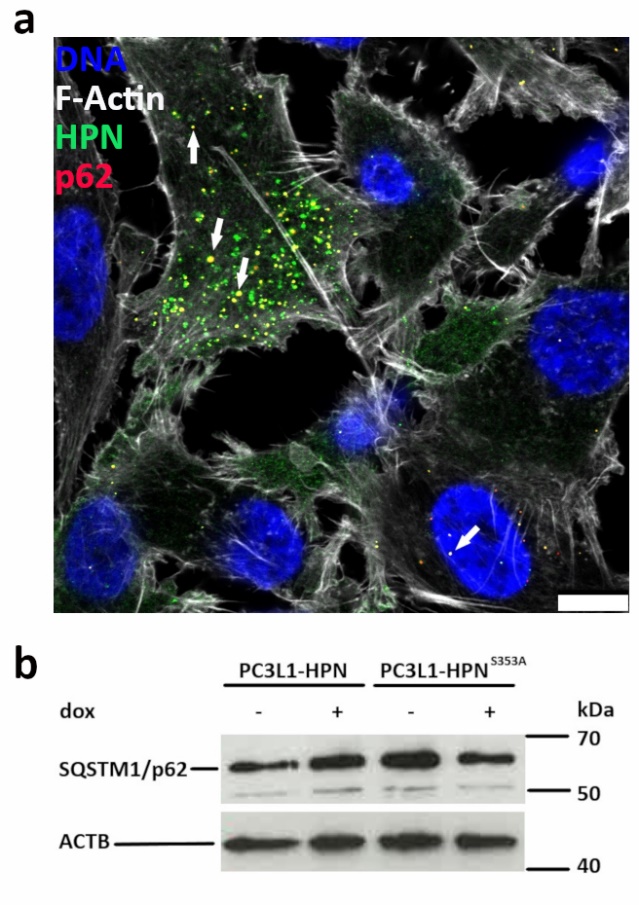


**Supplementary Figure 7:** (a) Immunofluorescence staining of hepsin and SQSTM1/p62 during overexpression of protease-deficient hepsin in PC3L1-HPN^S353A^ cells induced with dox (100 pg/ml, 48 h). Co-localization of both proteins is indicated by yellow color and exemplarily marked by arrows (scale bar: 10 µm). (b) Western blot analysis of SQSTM1/p62 protein expression in PC3L1-HPN and PC3L1-HPN^S353A^ in absence or presence (100 pg/ml, 48 h) of dox as indicated. ACTB was probed as a control for equal protein loading.

##
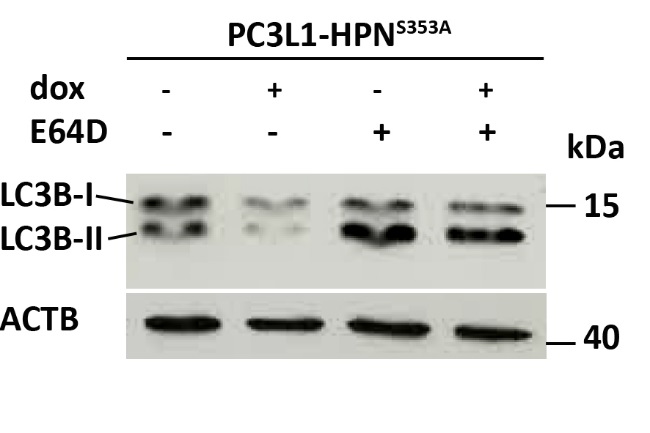


**Supplementary Figure 8:** Western blot analysis of LC3B protein expression (upper band: LC3B-I, lower band: LC3B-II) in PC3L1-HPN^S353A^ cell populations treated with E64D (10 µM, 24h) in absence or presence (100 pg/ml, 48 h) of dox as indicated. ACTB was probed as a control for equal protein loading.

**
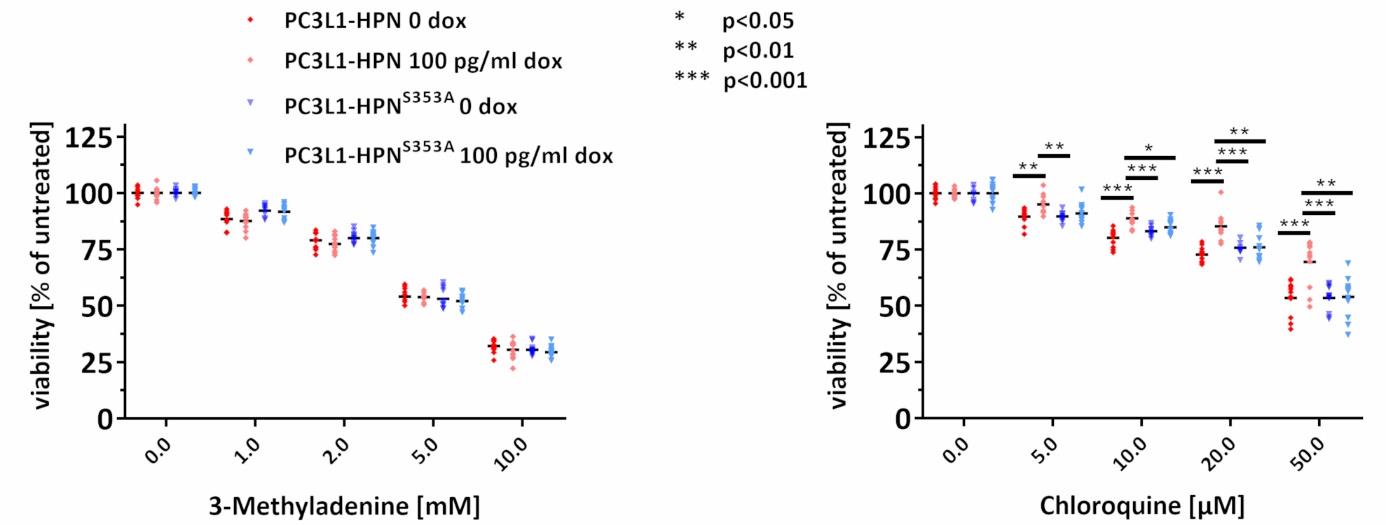
**

**Supplementary Figure 9:** Viability of PC3L1-HPN and PC3L1-HPNS353A in response to 24 h treatment with different concentrations of 3-Methyladenine (left diagram) and 48 h Chloroquine (right diagram) at different expression levels of the transgene as indicated. The graph shows mean viability + standard deviation (SD) as percentages of the untreated/uninduced control, respectively. Four independent experiments were performed in triplicates (n=12).


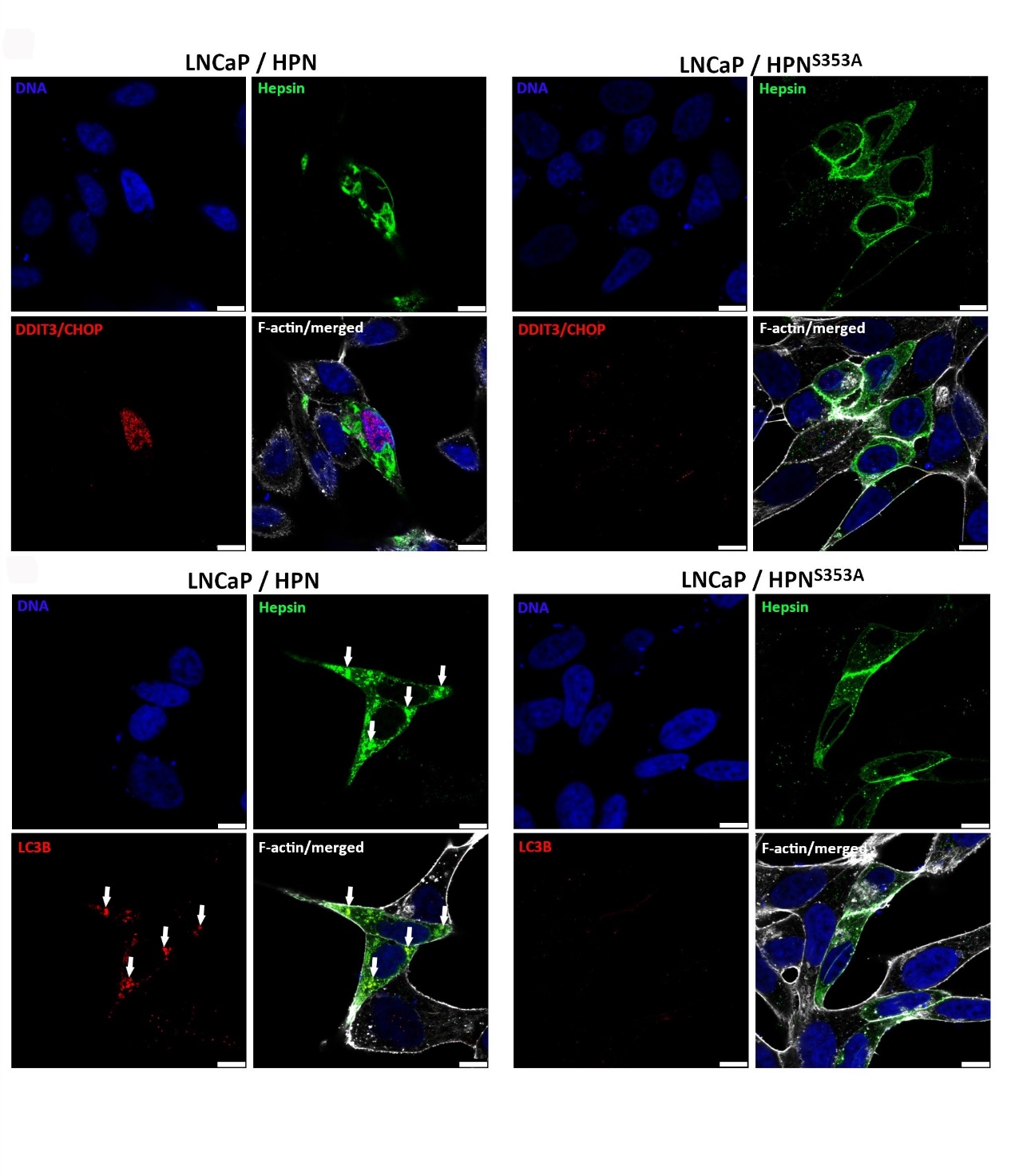


**Supplementary Figure 10:** Immunofluorescence analyses of the expression of DDIT3/CHOP (upper panel) and LC3B (lower panel) in LNCaP cell populations transiently transfected with wild-type (HPN, left column) and protease-deficient hepsin (HPN^S353A^, right column). The exemplary images show mixed populations of cells exhibiting either absence or high level expression of the respective hepsin transgene. Nuclear localization of DDIT3/CHOP could be frequently observed in cells overexpressing wild-type hepsin, but only rarely in cells overexpressing protease-deficient hepsin. LC3B-foci could be frequently observed in cells overexpressing wild-type hepsin, but only rarely in cells overexpressing protease-deficient hepsin. Co-localization of LC3B and wild-type hepsin is exemplarily indicated by arrows (scale bar: 10 µm).


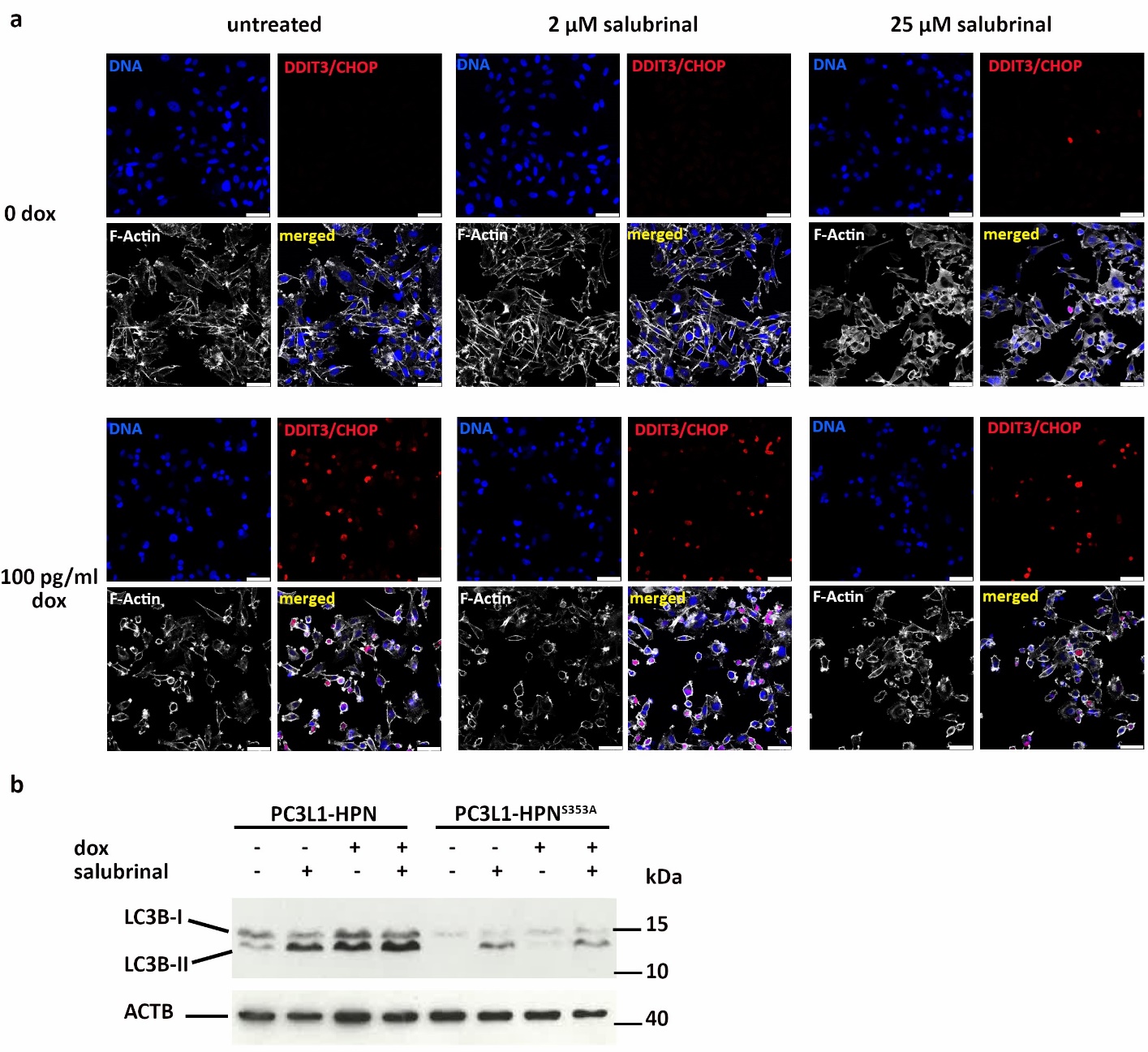


**Supplementary Figure 11:** (a) Immunofluorescence analyses of the expression of DDIT3/CHOP in PC3L1-HPN cell populations in absence (upper panel) and presence (lower panel) of dox subsequent to treatment with different doses of salubrinal for 48 hours as indicated. Nuclear expression of DDIT3/CHOP correlated with induction of hepsin and was not affected by salubrinal treatment (scale bar: 50 µm). (b) Western blot analysis of LC3B protein expression (upper band: LC3B-I, lower band: LC3B-II) in PC3L1-HPN and PC3L1-HPN^S353A^ cell populations treated with salubrinal (10 µM, 48h) in absence or presence (100 pg/ml) of dox as indicated. ACTB was probed as a control for equal protein loading.


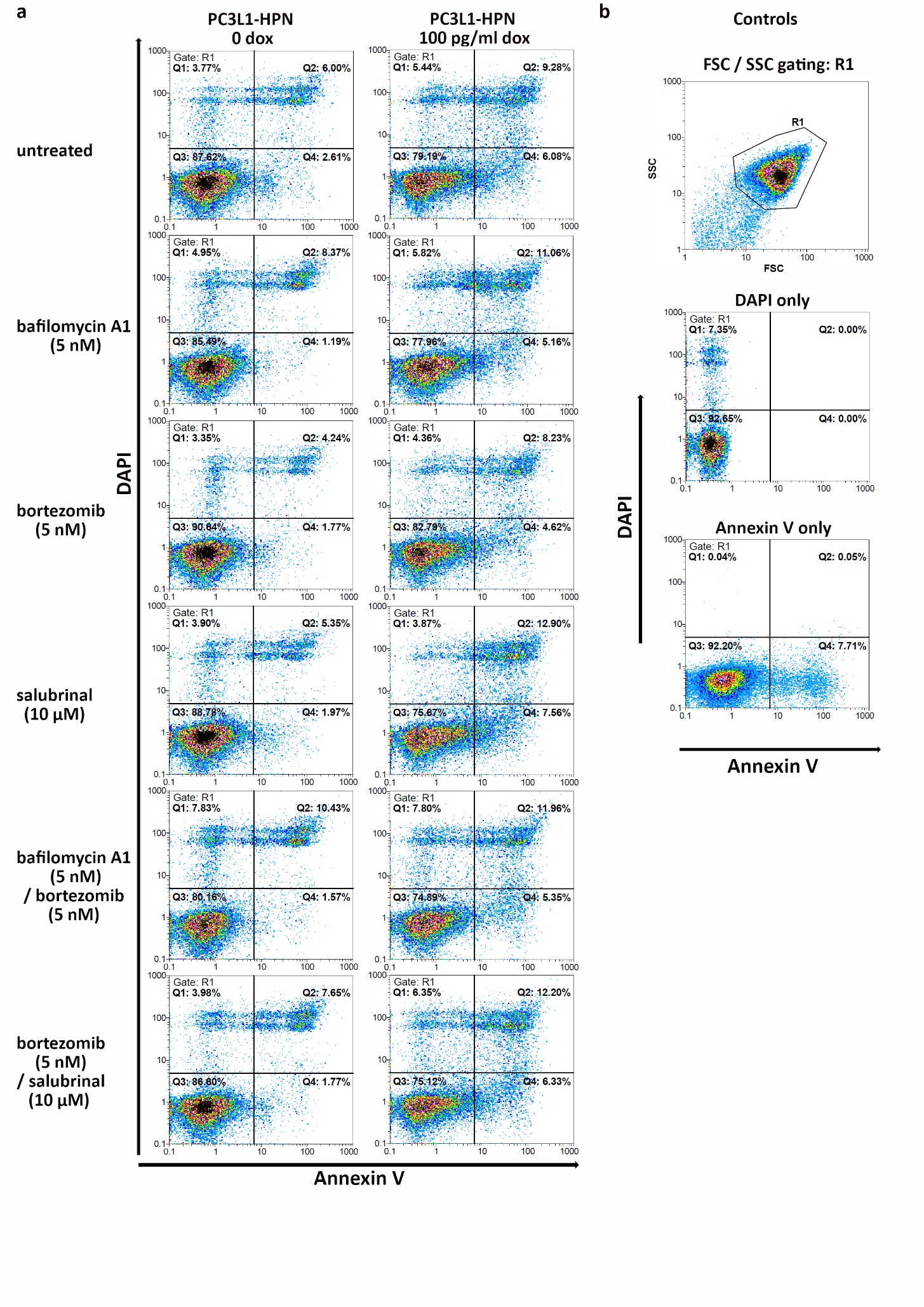


**Supplementary Figure 12:** Evaluation of apopotosis in PC3L1-HPN in the absence and presence of dox (100 pg/ml) and inhibitory compounds. Cells were seeded in presence (100 pg/ml) and absence of dox and grown for 48 h, followed by treatment with inhibitors as indicated. Twenty-four hours post inhibitor treatment, cells were stained with Annexin V-FITC and DAPI and subjected to flow cytometry. (a) Quadrant analysis of differentially treated cell populations from one of two experiments yielding similar results. Prior to quadrant analysis, cell populations were gated basing on their scattering characteristics (R1, exemplarily shown in b, upper image). The settings for quadrant analysis were defined using control cell populations (PC3L1 0 dox / untreated) which were negative for one fluorescent stain, respectively (b, lower images). The figure shows one out of three experiments with similar results.
